# Internal Vibrations of Pyridinium Cation in One-Dimensional Halide Perovskites and the Corresponding Halide Salts

**DOI:** 10.3390/molecules29010078

**Published:** 2023-12-22

**Authors:** Anna Yu. Samsonova, Alena Yu. Mikheleva, Kirill M. Bulanin, Nikita I. Selivanov, Anton S. Mazur, Peter M. Tolstoy, Constantinos C. Stoumpos, Yury V. Kapitonov

**Affiliations:** 1 Photonics of Crystals Laboratory, Saint Petersburg State University, Ulyanovskaya d.1, 198504 St. Petersburg, Russia; 2 Magnetic Resonance Research Center, Saint Petersburg State University, Universitetskiy pr. 26, 198504 St. Petersburg, Russia; a.mazur@spbu.ru; 3 Institute of Chemistry, Saint Petersburg State University, Universitetskiy pr. 26, 198504 St. Petersburg, Russia; 4 Department of Materials Science and Technology, University of Crete, Voutes, GR-70013 Heraklion, Greece

**Keywords:** halide perovskites, Raman scattering, IR absorption, solid-state NMR, molecular vibrations

## Abstract

We investigate vibrations of the pyridinium cation PyH^+^ = C_5_H_5_NH^+^ in one-dimensional lead halide perovskites PyPbX_3_ and pyridinium halide salts PyHX (X^−^ = I^−^, Br^−^), combining infrared absorption and Raman scattering methods at room temperature. Internal vibrations of the cation were assigned based on density functional theory modeling. Some of the vibrational bands are sensitive to perovskite or the salt environment in the solid state, while halide substitution has only a minor effect on them. These findings have been confirmed by ^1^H, ^13^C and ^207^Pb solid-state nuclear magnetic resonance (NMR) experiments. Narrower vibrational bands in perovskites indicate less disorder in these materials. The splitting of NH-group vibrational bands in perovskites can be rationalized the presence of nonequivalent crystal sites for cations or by more exotic phenomena such as quantum tunneling transition between two molecular orientations. We have shown how organic cations in hybrid organic–inorganic crystals could be used as spectators of the crystalline environment that affects their internal vibrations.

## 1. Introduction

The last decade has witnessed an increase in the interest in hybrid organic–inorganic lead-based halide perovskites due to their optoelectronic and photovoltaic applications [1,2,3,4]. This family of materials is valuable due to the wide variety of crystal structures, the most studied and used of which are three-dimensional (3D) lead halide perovskites with the chemical formula APbX_3_ (X^−^ = I^−^, Br^−^, Cl^−^). The 3D crystal framework is formed by the corner-shared lead-halide octahedra and small organic cations A^+^ between them (A^+^ = CH_3_NH_3_^+^ = MA^+^, HC(NH_2_)^+^ = FA^+^). Substitution of the organic cation A^+^ by a larger cation leads to the formation of low-dimensional perovskite-like structures, consisting of two-dimensional (2D) sheets [5,6] or one-dimensional (1D) chains [7,8] of lead-halide octahedra and the organic cations filling the space between them. Further, for brevity, such structures are designated as low-dimensional perovskites.

The movement of organic cations in hybrid lead halide perovskites and their low-dimensional analogues is the key to understanding the features of the crystal structure and its phase transitions. In case of 3D hybrid lead halide perovskites, order–disorder changes in the methylammonium (MA^+^) and formamidinium (FA^+^) cations were shown to be responsible for the distortion of the lead halide inorganic framework leading to the dynamic disorder at high temperatures [9,10,11,12,13]. MA^+^ and FA^+^ cations behave similarly in the high-temperature and intermediate-temperature phases, rotating isotropically and reorienting between their preferred orientations, respectively [9,14,15,16,17,18,19]. In the low-temperature phase, MA^+^ cations still have an ordered three-fold rotational axis [17,20], while the FA^+^ cations are locally disordered, exhibiting a glass-like state in the arrangement of the cations [14]. In 2D perovskites, large organic cations have greater freedom of motion compared to 3D perovskites and tend to interact with each other by weak intermolecular forces and with the inorganic framework through strong electrostatic interactions [5,6,21,22,23]. In addition, the motion of organic cations in these materials is responsible for order–disorder structural phase transitions with their symmetry breaking due to the concerted alignment of organic cations across a specific dipole moment vector direction [24,25,26]. For 1D hybrid halide perovskites, a major group of which are perovskites with face-shared octahedra assembled into the inorganic chains, organic cations are found to demonstrate behavior similar to that in their 2D counterparts [27,28,29,30].

To study the organic subsystem of hybrid halide perovskites, neutron [9] and X-ray diffraction (XRD) [9,10,24,25,26,27,28,29,30,31,32], solid-state nuclear magnetic resonance (NMR), quasielastic neutron scattering (QENS) [14,15,16,17], Raman [10,11,12,13,21,22,23,24,33] scattering methods and density functional theory (DFT) modeling [22,31,34] are widely used. The vibrational properties of inorganic perovskite frameworks have been studied in the literature [5,10,21,23]. Insufficient attention has been paid to the use of such relatively experimentally convenient methods as infrared (IR) and Raman spectroscopy for the study vibrations of the organic subsystem in perovskites. Organic cations are spectators of the crystalline environment [22,35]. The polarized Raman spectroscopy of organic vibrations could be used to determine the crystal orientation [33] and the cation movement [20].

Of particular interest is the movement of the simple aromatic pyridinium cation PyH^+^ = C_5_H_5_NH^+^, which stabilizes both perovskite-like compounds and PyHX (X^−^ = I^−^, Br^−^) salts [36,37,38,39,40]. The hybrid halide perovskites PyPbX_3_ (X^−^ = I^−^, Br^−^) have the 1D structure of face-shared octahedra chains [31,32,41,42].

In this work, we studied the vibrational properties of the pyridinium cation in hybrid halide perovskites PyPbX_3_ (X^−^ = I^−^, Br^−^). IR and Raman spectra of these perovskites are compared with ones for PyHX (X^−^ = I^−^, Br^−^) salts. Based on theoretical modeling, we identified the observed vibrations. Frequencies were identified that were most sensitive to changing the crystalline environment of the PyH^+^ cation from salt to perovskite, while replacing halogen usually had less effect. The latter statement is also confirmed by the solid-state NMR examination of organic cations. We also address the possible origins of the splitting of several bands involving cation NH-vibrations in perovskites.

## 2. Results and Discussion

### 2.1. Synthesis and Crystal Structure

Pyridinium lead trihalide PyPbX_3_ (X^−^ = I^−^, Br^−^) single crystals (see photos of the samples on Figure S1) were grown by the slow counterdiffusion of ions from individual solutions of lead(II) halides and pyridine in hydrohalic acid in the silica gel filled U-tube. Pyridine hydrohalides PyHX (X^−^ = I^−^, Br^−^) were synthesized by adding concentrated hydrogen halogenides drop-wise with constant stirring to the solutions of ethanol and pyridine. More details on the synthesis can be found in the Methods section (Section 3).

**Figure 1:**
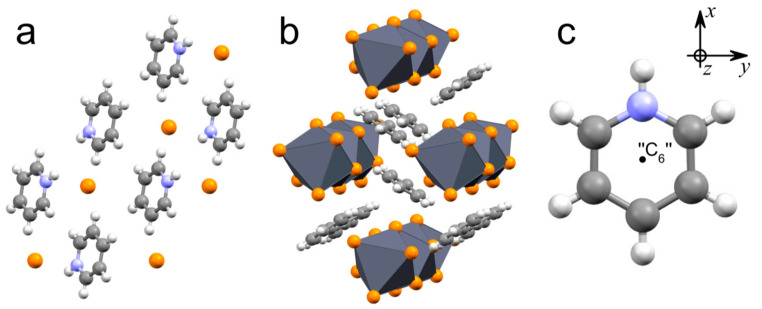
Typical crystal structure of PyHX [43,52] (**a**) and PyPbX_3_ [31,32,41,42] (X^−^ = I^−^, Br^−^) (**b**). (**c**) PyH^+^ cation. Gray—carbon atoms; blue—nitrogen atoms; white—hydrogen atoms; orange—halide atoms; dark grey—PbX_6_ octahedra.

The typical crystal structure of PyHX salts (X^−^ = I^−^, Br^−^) is shown in Figure 1a [43]. Upon heating from cryogenic to room temperature, these materials undergo an order–disorder phase transition from the monoclinic to the rhombohedral phase, in which the previously frozen PyH^+^ cations begin to rotate around the pseudo-six-fold axis (“C_6_”) (Figure 1c) [37,38,39]. The order–disorder phase transitions and molecular motions of the PyH^+^ cation in various compounds have been studied [36,37,38,39,40,44,45,46,47]. Pyridinium internal vibrational modes are shown to be sensitive to such phase transitions [48,49,50,51].

The crystal structure of PyPbX_3_ (X^−^ = I^−^, Br^−^) perovskites consists of 1D chains of face-shared lead halide octahedra and tightly packed PyH^+^ cations between them (Figure 1b) [31,32,41,42]. At room temperature, the compounds crystallize in the orthorhombic space group *Pnma* with each unit cell containing two chains running down the crystallographic *b*-axis and isolated from one another by individual PyH^+^ cations [32]. In the case of the simple salts, pyridinium cations can be found in a totally disordered state (PyHI, 293 K) [43] or a totally ordered state (PyHBr, 100 K) [52] depending on whether the intermolecular forces are strong enough to dominate over the thermal motion.

In both cases, the pyridinium cations remain essentially isolated from their surroundings. Thus, to analyze their internal vibrations, we will consider the vibrations of free cations as a first approximation and then estimate the influence of the crystalline environment on them. Another possible approach is to calculate the vibrations of cations in clusters, which are fragments of the crystal lattice [34].

### 2.2. Symmetry Considerations

To identify vibrations of pyridinium cations, we first consider the benzene (C_6_H_6_) molecule. We would like to note that the assignment of vibrational modes in the spectra of these molecules is a subject of discussion in the literature. To denote the vibrational modes of the PyH^+^ cation, we follow the Wilson notation [53], which was introduced to denote vibrational modes of the benzene C_6_H_6_ molecule and has been commonly used for the vibrational modes assignment of C_6_H_6_ derivatives [49,51,54].

The benzene C_6_H_6_ molecule belongs to the D_6h_ point group and contains a main C_6_ axis which contains S_6_ and S_3_ axes. Three C_2_ and three C_2′_ axes are perpendicular to the C_6_ axis and are passing through the middle of the bonds between carbon atoms and carbon atoms themselves, respectively. There are one σ_h_, three σ_v_ and three σ_d_ planes, an inversion center, as well as twelve irreducible representations (see the character table in Table S1).

The PyH^+^ cation (Figure 1c) can be considered as a C_6_H_6_ molecule in which one of the carbon atoms is replaced by a nitrogen atom. It leads to the loss of benzene symmetry operations except for the reflection in the one vertical plane σ_v_, rotation around the C_2′_ axis lying in the σ_v_ plane and reflection in the horizontal plane σ_h_ (Figure S2). These symmetry operations form the C_2v_ point group (see the character table in Table S2) and should be renamed as C_2′_ → C_2_, σ_h_ → σ_d_ and σ_v_ → σ_v_. The twelve irreducible representations of the D_6h_ point group are reduced to the four irreducible representations of the C_2v_ point group (Table S3), which are A_1_, A_2_, B_1_, B_2_. Here, we align the C_2_ axis with the *x*-axis in the Cartesian coordinate system. Indexes 1 and 2 denote the symmetry and asymmetry with respect to the reflection in the σ_v_ plane. Indexes A and B denote the symmetry and asymmetry with respect to the rotation around the C_2_ axis.

The C_6_H_6_ molecule and PyH^+^ cation both have *N* = 12 atoms and 3*N* = 36 degrees of freedom. In the case of an isolated cation or molecule, only the change in the position of the atoms relative to each other matters. Therefore, 36 degrees of freedom can be reduced by 6, of which 3 are translational movements and 3 are rotational movements of the molecule or cation as a whole. This results in 3*N* − 6 = 30 internal vibrational modes of an isolated C_6_H_6_ molecule or PyH^+^ cation. These modes are generated by one of the irreducible representations of the corresponding point symmetry group. The C_6_H_6_ molecule has doubly degenerate normal modes. For the PyH^+^ cation, this degeneracy is removed and resulting modes are denoted by indexes *a* or *b*. This notation was introduced by Wilson [53] and is widely used [54,55].

### 2.3. DFT Modeling

IR and Raman wavenumbers and intensities of internal vibrational modes of the single C_6_H_6_ molecule and PyH^+^ cation were calculated using DFT modeling (see Table 1). We use C_6_H_6_ modes notations recently refined by Gardner et al. [55] based on Wilson’s original work [53]. Correlating the vibrations of pyridinium with the established Wilson nomenclature for benzene requires care. We have assigned Wilson notation to pyridinium vibrational modes based on the correspondence between irreducible representations of the C_6_H_6_ molecule and PyH^+^ cation (Table S3), calculated IR and Raman intensities for both molecules (Table 1) and the motions of atoms (Figure S3). Closely lying vibrations with the same symmetries could interact and mix upon transition from benzene to pyridinium. We denote such modes with a dash. In the strict sense, these modes could not be considered as pure Wilson modes, but they are combinations of them. It also could be seen from the comparison of atomic motions in Figure S3 and in Wilson modes [53,55].

For some PyH^+^ cation vibrational modes, a substantial growth in activity is observed compared to those for the C_6_H_6_ molecule. Such modes are marked with an asterisk in Table 1. However, in several cases, the PyH^+^ cation mode inherits the activity of a vibrational mode of the C_6_H_6_ molecule, which was used as an additional criterion for mode designation. Next, the calculated pyridinium modes were correlated with the experimentally obtained ones.

### 2.4. Vibrational Spectra Summary

**Figure 2:**
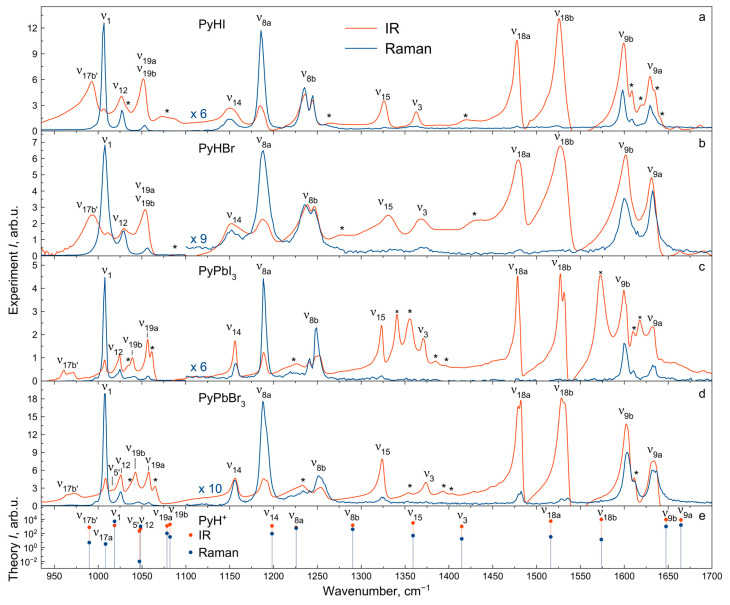
IR and Raman spectra of PyHX salts (**a**,**b**) and PyPbX_3_ perovskites (X^−^ = I^−^, Br^−^) (**c**,**d**) at *T* = 300 K; *—combination modes; ×—KI contamination bands (observed also in KI powder). (**e**) Calculated IR (red) and Raman (blue) intensities of PyH^+^ cation modes.

**Figure 3:**
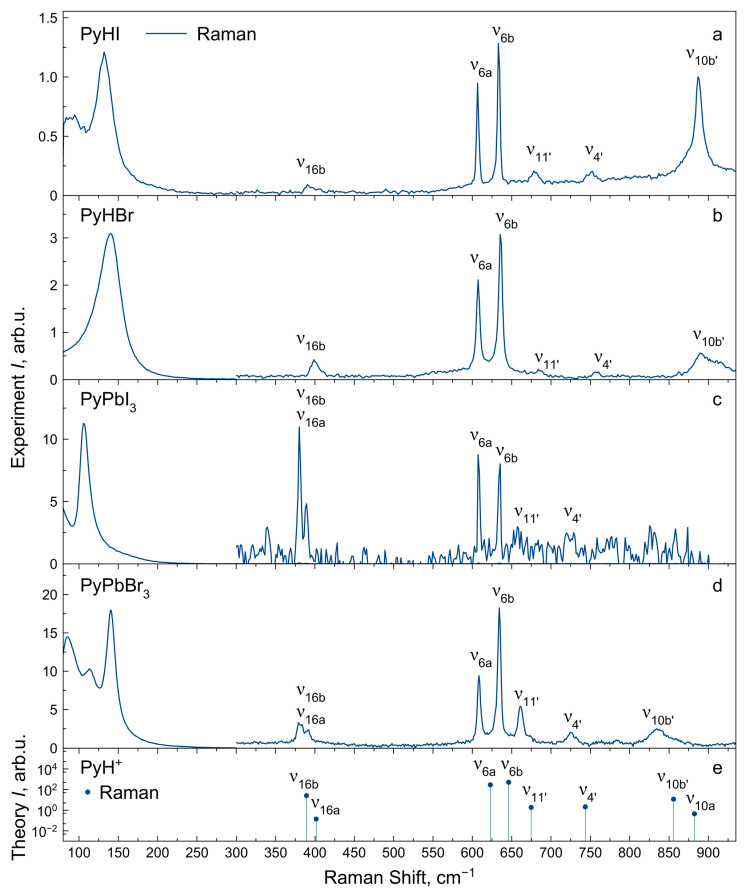
Raman spectra of PyHX salts (**a**,**b**) and PyPbX_3_ perovskites (X^−^ = I^−^, Br^−^) (**c**,**d**) at *T* = 300 K in the low-frequency region. (**e**) Calculated Raman intensities of PyH^+^ cation modes.

**Figure 4:**
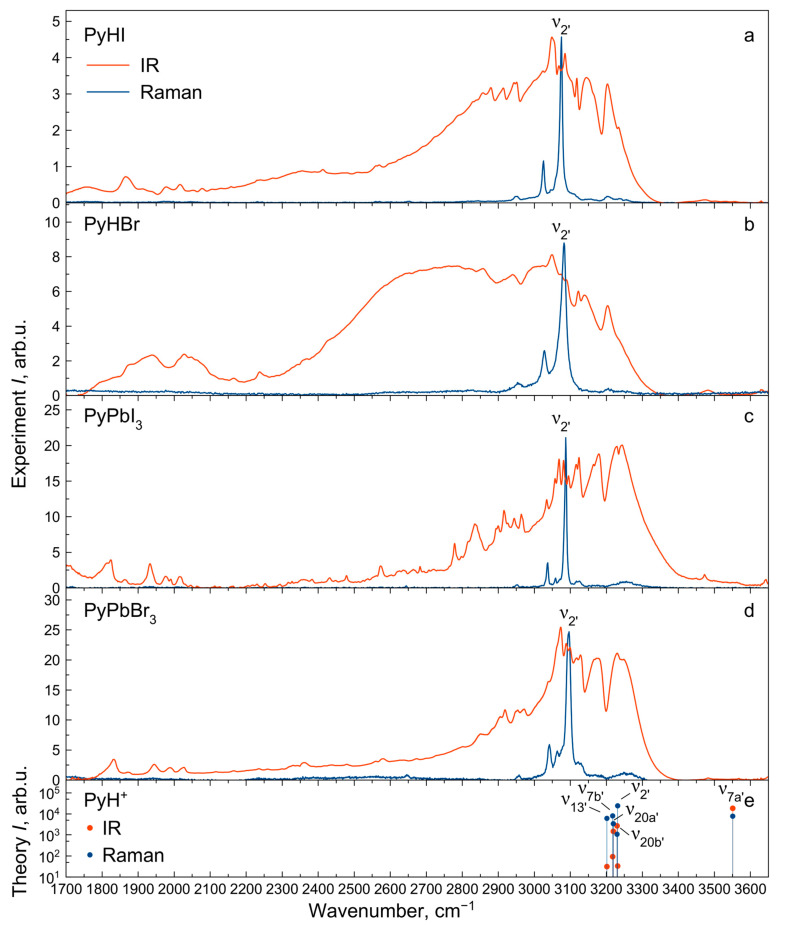
IR and Raman spectra of PyHX salts (**a**,**b**) and PyPbX_3_ perovskites (X^−^ = I^−^, Br^−^) (**c**,**d**) at *T* = 300 K in the high-frequency region. (**e**) Calculated IR (red) and Raman (blue) intensities of PyH^+^ cation modes.

The experimental IR and Raman spectra were measured for PyHX salts and PyPbX_3_ perovskites (X^−^ = I^−^, Br^−^) at room temperature (Figure 2, Figure 3 and Figure 4). The description of IR and Raman experiments could be found in the Methods section (Section 3). In this work, we focus on internal vibrations of the PyH^+^ cation lying above 350 cm^−1^. We will discuss three spectral regions: the low-frequency region 80–930 cm^−1^ (Figure 3, only Raman), medium-frequency region 930–1700 cm^−1^ with fingerprint vibrations (Figure 2, both IR and Raman) and high-frequency region of hydrogen modes 1700–3600 cm^−1^ (Figure 4, both IR and Raman). Correlating calculated cation vibrations with observed bands is a rather difficult task, but simultaneous analysis of IR and Raman spectra in four different materials makes this correlation more reliable. Below, we will consider in more detail the various spectral regions and give reasons for the chosen correlation presented in Table 1. Here, we adopted the notation of *ν*_8_, *ν*_9_, *ν*_14_, *ν*_15_, *ν*_18_ and *ν*_19_ modes of benzene from Gardner et al.’s work [55]. Notation in other works may differ [51,54].

Three vibration modes (*ν*_16a_, *ν*_10a_, and *ν*_17a_) have the A_2_ symmetry. The vibrations of this symmetry are forbidden in IR spectra. Calculations also showed that the Raman intensity of these modes is negligible. Therefore, these bands were not identified in the spectra. The intensities of unidentified vibrations in the experimental spectra are marked with the symbol “0” in Table 1.

### 2.5. Vibrational Spectra in Medium-Frequency Region

Let us consider the medium-frequency region 930–1700 cm^−1^ (Figure 2). The fully symmetric A_1_ vibrational modes *ν*_1_, *ν*_12_, *ν*_8a_, *ν*_18a_ and *ν*_9a_ are clearly recognized in all IR and Raman spectra with approximately the same frequencies in different compounds, which indicate that they are insensitive to the crystalline environment.

The doubly degenerate benzene mode *ν*_19_ splits into *ν*_19a_ and *ν*_19b_ modes of different symmetry in the pyridinium cation. The calculated splitting of these modes is 3.5 cm^−1^. This is quite consistent with the broad unresolved band observed in salts around 1050 cm^−1^. However, in perovskites, the crystal field causes a more significant splitting of these modes for around 7 cm^−1^ so that they could be potentially resolved in the IR spectra.

The vibrations *ν*_8b_, *ν*_18b_, *ν*_9b_ (mainly consisting of stretching ν_CN_ and in-plane bending δ_NH_ modes) and *ν*_19b_, *ν*_14_, *ν*_15_, *ν*_3_ (mainly consisting of stretching ν_CC_ and in-plane bending δ_CH_ modes) have the B_2_ symmetry. Except for the *ν*_19b_ vibration, all of them have the same frequency in the vibrational spectra of both perovskites and salts.

In the medium-frequency region, several overtones and combination modes of intense low-frequency vibrations are also observed. In order to identify these modes, the wavenumbers and intensities of normal modes and their combinations were refined by modeling in the anharmonic approximation (Table S4). The combination modes found in the spectra are summarized in Table S5 and marked by an asterisk in Figure 2.

For most of the modes with the A_1_ and B_2_ symmetries in this frequency range, no significant differences in position are observed for perovskites and salts. The *ν*_5′_ and *ν*_17b′_ vibrations (out-of-plane bending γ_NH_ and γ_CH_ modes, respectively) have B_1_ symmetry and low intensities in Raman spectra. The *ν*_5′_ vibrational band fully overlaps with other bands in the 1000–1050 cm^−1^ region of the IR spectra of salts. Narrower vibrational bands in the IR spectra of perovskites make it possible to assign the band at 1016 cm^−1^ to the *ν*_5′_ mode. In contrast, the frequency position of the *ν*_17b′_ vibration is clearly defined in the IR spectra. It significantly lowers its frequency for perovskites compared to salts (from 993 to 969 cm^−1^) and changes its shape. For PyHX (X^−^ = I^−^, Br^−^) salts, it has been noticed that there is a connection between the behavior of the vibrational band at 993 cm^−1^ and stretching ν_NH_ mode with the strength of the hydrogen bond [54]. The *ν*_17b′_ vibration frequency is lower in perovskites as a manifestation of hydrogen bond weakening in comparison to salts.

### 2.6. Vibrational Spectra in Low-Frequency Region

For the low-frequency region of 80–950 cm^−1^, only the Raman spectra were recorded (Figure 3). Experimental IR intensities of the vibrational bands in this region are marked with the symbol “-” in Table 1. The *ν*_10b′_, *ν*_4′_ and *ν*_11′_ vibrations with B_1_ symmetry in this region involve the γ_NH_ mode and lower the frequency in perovskites compared to salts. Accurate determination of the positions of these modes is important because they have high IR activity, which will lead to the appearance of overtones and combination modes in the spectrum. Measuring these modes with basic IR spectroscopy instruments is not possible. However, they are observed as weak lines in Raman spectra. Measured frequencies were used to determine spectral positions of overtones and combination frequencies in the spectrum.

The *ν*_6b_ (in-plane bending δ_CC_ mode) and *ν*_6a_ (out-of-plane bending γ_CC_ and γ_CN_ modes) can be clearly assigned in Raman spectra, and its wavenumbers are almost the same for salts and perovskites. The wavenumbers of *ν*_16b_ vibrations (out-of-plane bending γ_CC_ and γ_CN_ modes) can also be estimated for all compounds in Raman spectra, but the manifestation of *ν*_16a_ vibrations (in-plane bending δ_CC_ and δ_CN_ modes) in spectra of perovskites is uncertain.

The low-frequency intense band at 135 cm^−1^ in Raman spectra of halide salts is known as the rotational mode and does not depend on halide anion or hydrogen bond strength [56]. In perovskites, vibrations below 200 cm^−1^ correspond to the motion of the lead halide network. Its frequencies show dependency on halide atom substitution and are lower in PyPbI_3_ compared to PyPbBr_3_.

### 2.7. Vibrational Spectra in High-Frequency Region

The vibrational structure of the IR spectrum of halide salts and perovskites in the region of 1700–3400 cm^−1^ (Figure 4) is complex. Therefore, there is no accurate assumption of the stretching vibration frequency positions, which is marked with the symbol “?” in Table 1. The exceptions are the strongest vibrational band at 3075–3096 cm^−1^ in the Raman spectra (*ν*_2′_ mode) and the broadest vibrational band at 1700–3300 cm^−1^ in the IR spectra (*ν*_7a′_ mode).

The complexity of the IR spectrum of halide salts is explained by Fermi resonances of the stretching ν_NH_ mode (*ν*_7a′_) with overtones and combinations of PyH^+^ internal modes [56,57,58]. An increase in the frequency and a decrease in the intensity of the *ν*_7a′_ vibrational structure in the IR spectra indicates a significant hydrogen bond weakening in perovskites compared to salts. This trend and less intense and narrower Fermi resonances at the same frequencies also reveal a slight hydrogen bond weakening in PyPbI_3_ perovskite compared to PyPbBr_3_. The low-frequency component of the *ν*_7a′_ vibrational structure at 1700–2100 cm^−1^ (so-called “C-band”) [58] for PyPbBr_3_ perovskite is slightly shifted to the higher frequencies compared to PyPbI_3_, which is a manifestation of the higher vibrational coupling of the ν_NH_ mode with other modes in this spectral region. The bands at 1800–1880 cm^−1^, 1920–1990 cm^−1^ and 2010–2030 cm^−1^ are formed by the stretching ν_NH_ mode coupling with 2*ν*_10b′_, 2*ν*_17b′_ and 2*ν*_1_ modes, respectively. The higher frequency bands at 2880–3400 cm^−1^ are formed by the ν_NH_ mode coupling with 2*ν*_18a_, 2*ν*_18b_, 2*ν*_9b_, 2*ν*_9a_ modes and combinations of the last ones with lower modes.

For all IR and Raman spectra, narrower vibrational bands in the spectra of perovskites are observed compared to salts, which is due to the less disorder in perovskites. It can also be emphasized that most internal vibrational modes are not sensitive to the crystal structure (salt or perovskite) and halide anion (iodide or bromide). The exceptions are vibrational bands containing out-of-plane bending γ_NH_ (*ν*_11′_, *ν*_4′_, *ν*_10b′_, *ν*_17b′_, *ν*_5′_) and stretching ν_NH_ modes (*ν*_7a′_), which are shown to be strongly affected by the environment [48,49,50,51,54,55]. Thus, the observation of these modes can provide information about the crystal structure of the material.

### 2.8. Vibration Splitting Effect

**Figure 5:**
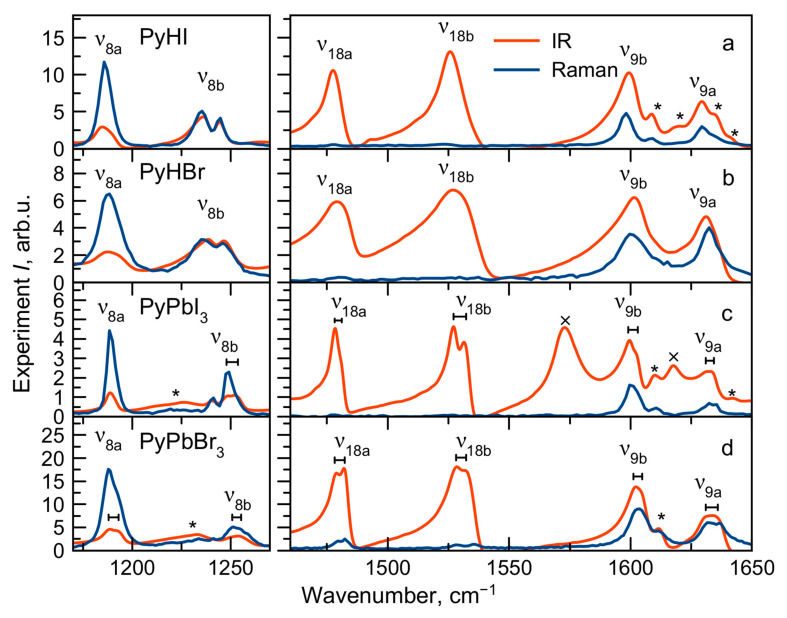
IR and Raman spectra of PyHX salts (**a**,**b**) and PyPbX_3_ perovskites (X^−^ = I^−^, Br^−^) (**c**,**d**) at *T* = 300 K in the region of split bands. *—combination modes; ×—KI contamination bands (observed also in KI powder).

The intriguing feature of PyPbX_3_ (X^−^ = I^−^, Br^−^) vibrational spectra is the splitting of the *ν*_8a_, *ν*_8b_, *ν*_18a_, *ν*_18b_, *ν*_9b_, *ν*_9a_ vibrational bands containing the in-plane δ_NH_ mode (Figure 5). Such an effect can originate from one of several sources: (i) intermolecular coupling of vibrations of molecules in equivalent positions in the unit cell at Z > 1 (Davydov splitting); (ii) the existence of two crystallographically non-identical cation positions in the unit cell (crystal cite effect); and (iii) the switching of cations between non-equivalent positions, leading to the splitting of involved vibrational modes.

The large interaction distance in the perovskite crystal structure speaks against the Davydov splitting effect. Splitting of the abovementioned bands was observed in the vibrational spectra of pyridinium salts of tungstophosphoric acid [59,60]. A possible explanation for this phenomenon is the mechanism of quantum tunneling of the cation between two positions, leading to the splitting of vibrations containing the NH-group bending mode. Possible mechanisms to explain the splitting observed in perovskites are the switching of pyridinium cation between non-equivalent positions and the presence of two nonequivalent crystal sites of pyridinium cations. Determining the specific nature of these splittings is possible by combining XRD and IR studies at lower temperatures. In hybrid organic–inorganic perovskites, at temperatures below room temperature, order–disorder phase transitions could be observed, caused by the “freezing out” of the movement of organic cations in the lattice. The corresponding change in the symmetry could be found from XRD data. A related issue is the presence of a pseudo two-fold axis passing through the center of C-N bond in the pyridinium cation in perovskite crystals at room temperature, which is a manifestation of the frustrated rotation of the cation between two symmetry equivalent positions.

### 2.9. Solid-State NMR Study

**Figure 6:**
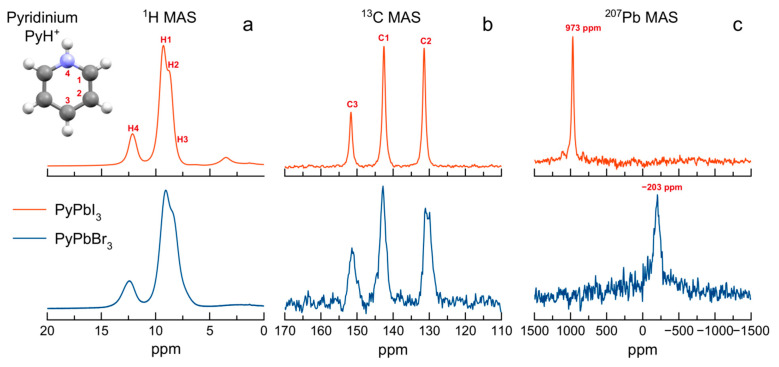
The ^1^H (one-pulse) (**a**), ^13^C (CP) (**b**) and ^207^Pb (Hahn-echo) (**c**) MAS (12.5 kHz) NMR spectra of PyPbI_3_ (red) and PyPbBr_3_ (blue) recorded at *T* = 298 K.

The ^1^H, ^13^C and ^207^Pb NMR spectra of PyPbX_3_ (X^−^ = I^−^, Br^−^) with the assignment of the signals are shown in Figure 6. There seems to be no significant difference in the state of the pyridinium cation in bromine- and iodine-based perovskite samples, except for a somewhat larger line width in the case of bromine-based perovskite, which is probably due to a larger number of structural defects in the crystalline sample. Nevertheless, in both cases, the spectra are reasonably well resolved, and only one set of relatively narrow ^1^H and ^13^C NMR signals is observed. The chemical shifts of ^207^Pb NMR signals are noticeably different from those of 3D perovskites. We speculate that the values around –200 ÷ –300 ppm for PyPbBr_3_ and around 900–1000 ppm for PyPbI_3_ could be indicative for the formation of 1D chains of lead-halide octahedra.

## 3. Methods

*Synthesis*. Pyridinium lead trihalide PyPbX_3_ (X^−^ = I^−^, Br^−^) single crystals were grown by the slow counterdiffusion of ions from individual solutions of lead(II) halides and pyridine in hydrohalic acid with 1 M concentrations in the silica gel filled U-tube. This counterdiffusion-in-gel crystallization (CGC) method could be used for synthesis of high quality hybrid organic–inorganic low-dimensional perovskites and 3D perovskites [61]. XRD data are presented in the Supplementary Materials (Figure S4). Pyridine hydrohalides PyHX (X^−^ = I^−^, Br^−^) were synthesized by adding concentrated hydrogen halogenides drop-wise with constant stirring to the solutions of 5 mL of ethanol and 1 mL of pyridine. The resulting solutions were kept at a temperature of 3–5 °C for 12 h, then filtrated and rinsed with ethanol and dried. More details on the PyHX synthesis could be found in Selivanov et al. [31].

*DFT Calculations*. IR and Raman spectra for the PyH^+^ cation and C_6_H_6_ molecule were simulated in the harmonic approximation using the Gaussian 16 software package. The wavenumbers and IR intensities of normal and combination modes were refined in the anharmonic approximation for PyH^+^ cation. For all calculations, the B3LYP functional and 6-311+G(d,p) basis set with one set of polarizing functions for heavy atoms (d-type) and hydrogen (p-type) were used.

*Raman Measurements.* The experimental unpolarized Raman spectra of PyHX and PyPbX_3_ (X^−^ = I^−^, Br^−^) were acquired at *T* = 300 K using the Horiba Jobin-Yvon LabRam HR800 confocal Raman spectrometer (Horiba Jobin-Yvon, Oberursel, Germany) in backscattering geometry (see Figure S5 for the scheme of the Raman measurements). The equipment used a diffraction grating with a cell of 1800 L/mm and the aperture was 150 × 150 μm. Laser radiation was focused through a 100× objective lens. A solid-state laser with a wavelength of 532 nm was utilized as the radiation source. The actual laser power applied to the sample was around 6 mW, with 10 s of accumulation time and 6 repetitions.

*IR Measurements.* PyHX and PyPbX_3_ crystals were subjected to grinding in a mortar and mixed with KX powders (where X^−^ = I^−^, Br^−^) in a weight ratio of 1:10 for I and 1:3 for Br, respectively. IR absorption spectra were recorded in the 850–4000 cm^−1^ spectral range with a spectral resolution of 2 cm^−1^ applying the Happ–Gensel apodization using a research-grade FT-IR spectrometer Thermo Nicolet iS50 (Thermo Scientific, Waltham, MA, USA). Spectrometer was equipped with a DTGS detector and a KBr beamsplitter.

*Solid-state NMR Measurements.* The ^1^H, ^13^C and ^207^Pb NMR measurements were performed using Bruker Avance III 400WB NMR spectrometers (Bruker, Ettlingen, Germany) (working frequency 400.23 MHz for ^1^H, 100.65 MHz for ^13^C and 83.73 MHz for ^207^Pb). The spectra were recorded under magic angle spinning conditions (MAS; spinning rate 12 kHz) at room temperature using 4.0 mm rotor. ^13^C MAS NMR spectra were measured using cross-polarization (CP) technique (2 ms contact time); ^207^Pb NMR spectra were measured using Hahn echo technique. The relaxation delays were set to 120 s for ^1^H, 2–5 s for ^13^C and 1 s for ^207^Pb. Liquid TMS at 0 ppm was used as an external reference for ^1^H and ^13^C spectra. Pb(NO_3_)_2_ at −3482 ppm (under 4 kHz spinning with 25 °C room temperature) was used as an external reference for ^207^Pb spectra.

## 4. Conclusions

We conducted a study of the internal vibrations of the pyridinium cation PyH^+^ in four materials: PyHX salts and PyPbX_3_ (X^−^ = I^−^, Br^−^) 1D-perovskites. The bands experimentally observed in the Raman scattering and IR absorption spectra were identified by analyzing the symmetry of vibrations and comparing them with the results of DFT calculations for a free benzene molecule and a pyridinium cation. Most internal vibrations of PyH^+^ are not significantly influenced by the crystalline environment. However, vibrational modes *ν*_11′_, *ν*_4′_, *ν*_10b′_, *ν*_17b′_, *ν*_5′_ and *ν*_7a′_ undergo strong shifts upon the transition from salts to perovskites. What these modes have in common is the presence of NH-group movements in them, which, apparently, are influenced by the environment, in particular, by the changes in the strength of the hydrogen bond. In perovskites, splitting of non-degenerate modes *ν*_8a_, *ν*_8b_, *ν*_18a_, *ν*_18b_, *ν*_9b_, *ν*_9a_ is observed. This phenomenon can be explained by different mechanisms, which could be distinguished by a joint XRD and IR study at lower temperatures. This work illustrates the different crystalline environment influence on organic cations behavior such as vibrational band shifts, changes in their intensities and width and splitting of non-degenerate levels. In this way, organic cations act as spectators, probes of the crystalline environment in hybrid organic–inorganic crystals, including halide perovskites, and provide valuable information on the dynamics of the system. Thus, spectroscopy of internal vibrations of organic cations can be a convenient and informative tool for studying these new materials towards new photonics applications.

## Figures and Tables

**Table 1:** Calculated internal vibrational modes of C_6_H_6_ molecule and PyH^+^ cation, and experimental bands for pyridinium salts and perovskites.

Benzene C_6_H_6_ (Calculated)					Pyridinium PyH^+^ (Calculated)					Experiment, Freq., cm^−1^ (IR,R)			
	Sym.	Freq., cm^−1^	*I*_th_IR, arb.u.	*I*_th_R, arb.u.		Sym.	Freq., cm^−1^	*I*_th_IR, arb.u.	*I*_th_R, arb.u.	PyHI	PyHBr	PyPbI_3_	PyPbBr_3_
*ν*_16_	E_2u_	410.06	0.0	0.0	*ν*_16b_	B_1_	389.19	0.851	0.269	390 (-,w)	399 (-,w)	380 (-,s)	379 (-,w)
					*ν*_16a_	A_2_	401.53	0.0	0.001	(-,0)	(-,0)	(-,0)	(-,0)
*ν*_6_	E_2g_	622.21	0.0	4.718	*ν*_6a_	A_1_	622.88	0.014	2.883	607 (-,s)	607 (-,s)	607 (-,s)	608 (-,s)
					*ν*_6b_	B_2_	646.15	0.398	5.125	633 (-,s)	635 (-,s)	635 (-,s)	634 (-,s)
*ν*_11_	A_2u_	686.61	725.940	0.0	*ν*_11′_	B_1_	674.76	92.175	0.019	681 (-,w)	685 (-,w)	657 (-,w)	662 (-,w)
*ν*_4_	B_2g_	718.77	0.0	0.001	*ν*_4′_	B_1_	743.62	88.368 *	0.022	753 (-,w)	757 (-,w)	726 (-,w)	727 (-,w)
*ν*_10_	E_1g_	862.47	0.0	0.977	*ν*_10b__′_	B_1_	855.74	6.213 *	0.119	887 (-,s)	891 (-,w)	(-,0)	835 (-,w)
					*ν*_10a_	A_2_	882.32	0.0	0.005	(-,0)	(-,0)	(-,0)	(-,0)
*ν*_17_	E_2u_	987.00	0.0	0.0	*ν*_17b__′_	B_1_	989.76	1.795	0.049	993 (s,0)	993 (s,0)	968 (s,0)	971 (s,0)
					*ν*_17a_	A_2_	1008.38	0.0	0.031	(0,0)	(0,0)	(0,0)	(0,0)
*ν*_1_	A_1g_	1011.61	0.0	98.763	*ν*_1_	A_1_	1018.61	3.442 *	52.390	1007 (s,s)	1008 (s,s)	1008 (s,s)	1008 (s,s)
*ν*_5_	B_2g_	1016.96	0.0	0.0	*ν*_5′_	B_1_	1046.99	0.573	0.0	(0,0)	(0,0)	(0,0)	1016 (m,0)
*ν*_12_	B_1u_	1022.46	0.0	0.0	*ν*_12_	A_1_	1048.31	1.280	10.132 *	1027 (s,s)	1028 (s,s)	1025 (s,s)	1026 (s,s)
*ν*_19_	E_1u_	1059.14	24.137	0.0	*ν*_19a_	A_1_	1078.43	3.178	1.006	1052 (s,s)	1054 (s,s)	1058 (s,m)	1058 (s,m)
					*ν*_19b_	B_2_	1081.89	5.159	0.317			1041 (s,m)	1046 (s,m)
*ν*_14_	B_2u_	1174.67	0.0	0.0	*ν*_14_	B_2_	1198.20	3.550	0.913	1150 (s,s)	1151 (s,s)	1156 (s,s)	1158 (s,s)
*ν*_8_	E_2g_	1197.33	0.0	5.225	*ν*_8a_	A_1_	1225.74	2.207	5.432	1186 (s,s)	1188 (s,s)	1188 (s,s)	1189 (s,s)
													1193 (s,s)
					*ν*_8b_	B_2_	1290.23	4.616	3.836	1236 (s,s)	1238 (s,s)	1240 (s,s)	1252 (w,s)
										1245 (s,s)	1247 (s,s)	1248 (s,s)	1258 (w,s)
*ν*_15_	B_2u_	1337.39	0.0	0.0	*ν*_15_	B_2_	1359.06	10.665 *	0.483	1327 (s,w)	1330 (s,w)	1323 (s,w)	1324 (s,w)
*ν*_3_	A_2g_	1380.96	0.0	0.0	*ν*_3_	B_2_	1414.57	3.511	0.166	1364 (s,w)	1367 (s,w)	1371 (s,0)	1375 (s,0)
*ν*_18_	E_1u_	1510.48	18.87	0.0	*ν*_18a_	A_1_	1516.10	21.546	0.322	1478 (s,w)	1480 (s,w)	1478 (s,0)	1479 (s,w)
													1482 (s,w)
					*ν*_18b_	B_2_	1573.78	39.154	0.135	1526 (s,w)	1527 (s,0)	1527 (s,0)	1529 (s,s)
												1532 (s,0)	1536 (s,s)
*ν*_9_	E_2g_	1634.35	0.0	12.694	*ν*_9b_	B_2_	1647.59	38.561 *	9.756	1602 (s,s)	1600 (s,s)	1600 (s,s)	1603 (s,s)
					*ν*_9a_	A_1_	1664.58	34.814 *	15.265	1629 (s,s)	1632 (s,s)	1632 (s,w)	1633 (s,s)
												1635 (s,w)	1637 (s,s)
*ν*_13_	B_1u_	3156.58	0.0	0.0	*ν*_13′_	A_1_	3201.51	0.254	60.939 *	?	?	?	?
*ν*_7_	E_2g_	3166.22	0.0	133.318	*ν*_7b__′_	B_2_	3217.55	0.758	79.950	?	?	?	?
					*ν*_7a__′_	A_1_	3550.84	163.241 *	77.094	2934 ** (s,?)	2818 ** (s,?)	3102 ** (s,?)	3064 ** (s,?)
*ν*_20_	E_1u_	3181.82	47.147	0.0	*ν*_20a__′_	A_1_	3219.39	11.864	33.911 *	?	?	?	?
					*ν*_20b__′_	B_2_	3229.71	21.724	10.687 *	?	?	?	?
*ν*_2_	A_1g_	3192.01	0.0	421.879	*ν*_2′_	A_1_	3231.40	0.273	240.672	3075 (?,s)	3084 (?,s)	3087 (?,s)	3096 (?,s)

The table is sorted by calculated frequencies of benzene. Symbols: ′—mixed modes; *—modes with substantial growth in activity in PyH^+^ in comparison with C_6_H_6_; **—modes position calculated as center-of-mass of the broad band; s—strong band; w—weak band; 0—vibration is not observed in the experiment; -—no data for this region; ?—unable to determine frequency.

## Data Availability

Data are contained within the article and Supplementary Materials.

## Supplementary Materials

The following supporting information can be downloaded at: https://www.mdpi.com/article/10.3390/molecules29010078/s1, Figure S1: Photos of PyPbI_3_ (a) and PyPbBr_3_ (b) single crystals; Table S1: Character table of D_6h_ point symmetry group; Figure S2: Transformation of C_6_H_6_ to PyH^+^ by symmetry elements; Table S2: Character table of C_2v_ point symmetry group; Table S3: Correspondence between the irreducible representations of the D_6h_ and C_2v_ point symmetry groups; Figure S3: Calculated atomic displacements in internal normal modes of the PyH^+^ cation. The notation in Wilson nomenclature and symmetry of the modes are labeled. Red crosses and dots indicate out-of-plane atomic displacements; Table S4: Calculated anharmonic fundamental modes (from 1 to 30), overtones (Over) and combination modes (Comb) of the PyH^+^ cation; Table S5: Combination modes recognized in the experimental vibrational spectra; Figure S4: Powder XRD patterns simulated from the single crystal data (a,c) and measured for grounded single crystals (b,d) for PyPbI_3_ (a,b) and PyPbBr_3_ (c,d); Figure S5: The scheme of the Raman measurement experiment.
