# Supplementary material for: Internal Vibrations of Pyridinium Cation in One-Dimensional Halide Perovskites and the Corresponding Halide Salts
Source: Molecules. 2023 Dec 22;29(1):78. doi: 10.3390/molecules29010078 (PMC10780259; doi:10.3390/molecules29010078)
Supplement: Supplementary file 1 [file molecules-29-00078-s001.zip › molecules-2778389-supplementary.pdf]

Internal Vibrations of Pyridinium Cation in One-Dimensional Halide Perovskites and the Corresponding Halide Salts

Anna Yu. Samsonova[1,*], *Alena Yu. Mikheleva,*[1] *Kirill M. Bulanin,* [1] *Nikita I. Selivanov,* [1] *Anton S. Mazur,*[2] *Peter M. Tolstoy,*[3] *Constantinos C. Stoumpos* [1,4,*] *and Yury V. Kapitonov* [1]

1) Photonics of Crystals Laboratory, Saint Petersburg State University, Ulyanovskaya d.1, 198504, St. Petersburg, Russia

2) Magnetic Resonance Research Center, Saint Petersburg State University, Universitetskiy pr. 26, 198504 St. Petersburg, Russia

3) Institute of Chemistry, Saint Petersburg State University, Universitetskiy pr. 26, 198504 St. Petersburg, Russia

4) Department of Materials Science and Technology, University of Crete, Voutes, GR-70013 Heraklion, Greece

Email: a.y.samsonova@spbu.ru, cstoumpos@materials.uoc.gr

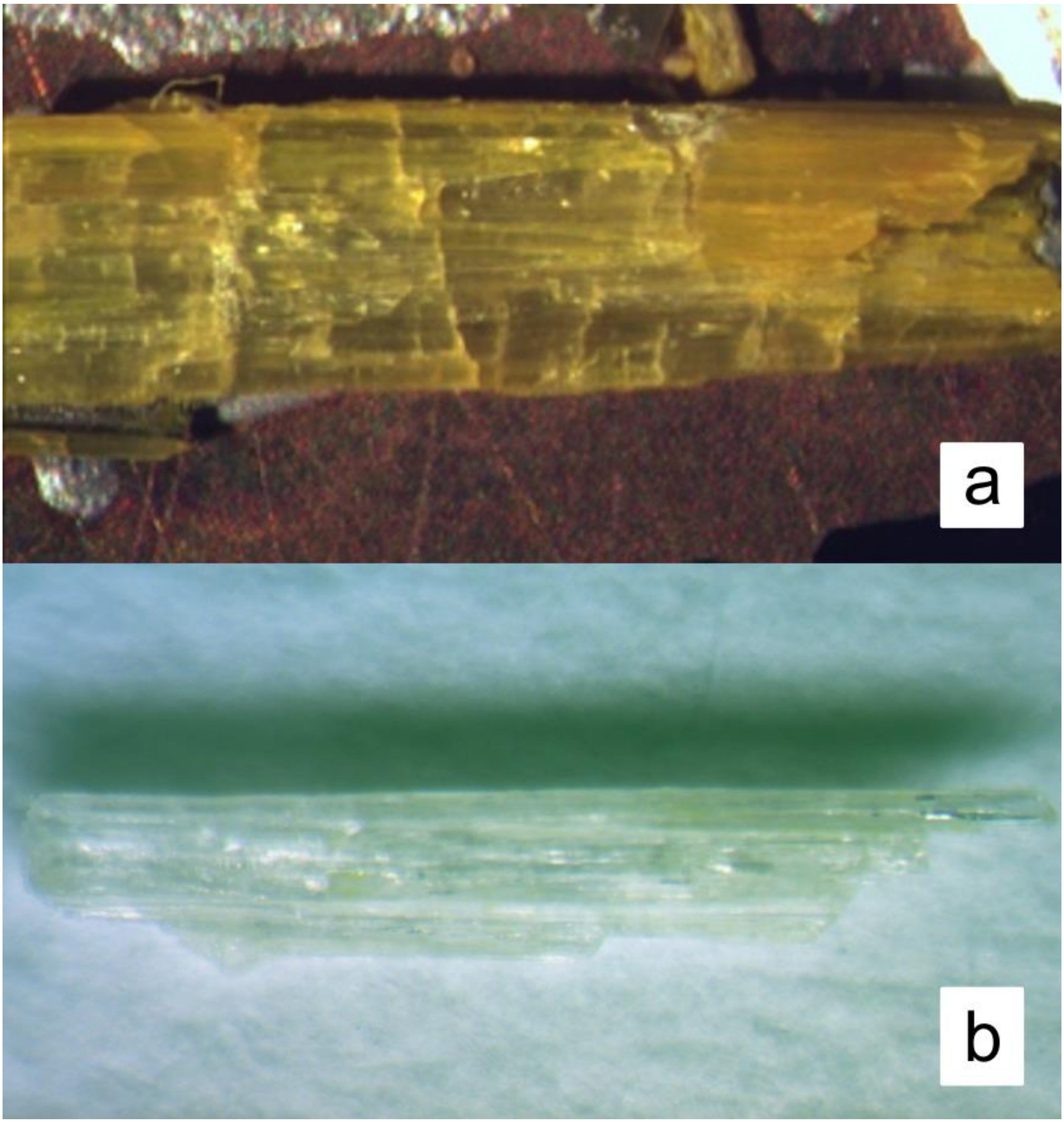

Figure S1. Photos of $PyPbI_3$ (a) and $PyPbBr_3$ (b) single crystals.

Table S1. Character table of D_{6h} point symmetry group.

D_{6h}	E	$2C_6$	$2C_3$	C_2	$3C_2'$	$3C_2''$	i	$2S_6$	$2S_3$	σ_h	$3\sigma_v$	$3\sigma_d$	IR	R
A_{1g}	+1	+1	+1	+1	+1	+1	+1	+1	+1	+1	+1	+1	-	+
A_{2g}	+1	+1	+1	+1	–1	–1	+1	+1	+1	+1	–1	–1	-	-
B_{1g}	+1	–1	+1	–1	+1	–1	+1	+1	–1	–1	–1	+1	-	-
B_{2g}	+1	–1	+1	–1	–1	+1	+1	+1	–1	–1	+1	–1	-	-
E_{1g}	+2	+1	–1	–2	0	0	+2	–1	+1	–2	0	0	-	+
E_{2g}	+2	–1	–1	+2	0	0	+2	–1	–1	+2	0	0	-	+
A_{1u}	+1	+1	+1	+1	+1	+1	–1	–1	–1	–1	–1	–1	-	-
A_{2u}	+1	+1	+1	+1	–1	–1	–1	–1	–1	–1	+1	+1	+	-
B_{1u}	+1	–1	+1	–1	+1	+1	–1	–1	+1	+1	+1	–1	-	-

B_{2u}	+1	–1	+1	–1	–1	–1	–1	–1	+1	+1	–1	+1	-	-
E_{1u}	+2	+1	–1	–2	0	0	–2	+1	–1	+2	0	0	+	-
E_{2u}	+2	–1	–1	+2	0	0	–2	+1	+1	–2	0	0	-	-

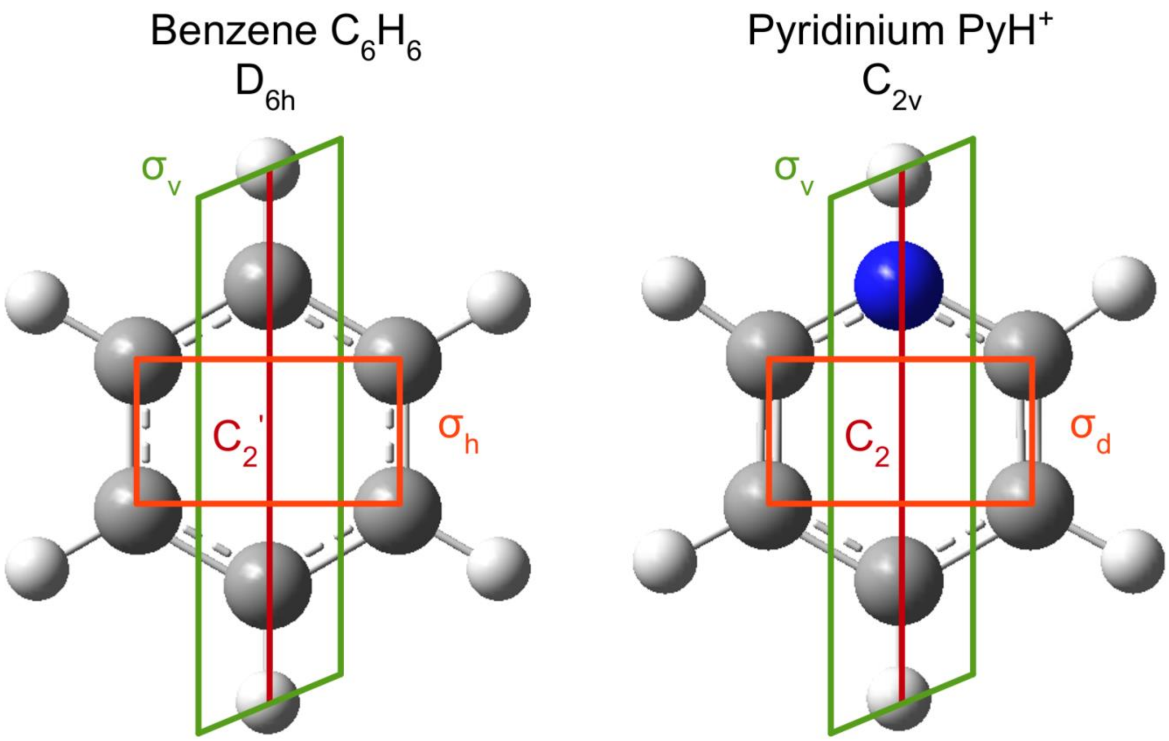

Figure S2. Transformation of C_6H_6 to PyH^+ by symmetry elements.

Table S2. Character table of C_{2v} point symmetry group.

C_{2v}	E	C_2	σ_v	σ_d	IR	R
A_1	+1	+1	+1	+1	+	+
A_2	+1	+1	–1	–1	-	+
B_1	+1	–1	+1	–1	+	+
B_2	+1	–1	–1	+1	+	+

Table S3. Correspondence between the irreducible representations of the D_{6h} and C_{2v} point symmetry groups.

D_{6h}	C_{2v}
A_{1g}	A_1
A_{2g}	B_2
B_{1g}	A_2
B_{2g}	B_1
E_{1g}	A_2+B_1
E_{2g}	A_1+B_2
A_{1u}	A_2

A_{2u}	B_1
B_{1u}	A_1
B_{2u}	B_2
E_{1u}	A_1+B_2
E_{2u}	A_2+B_1

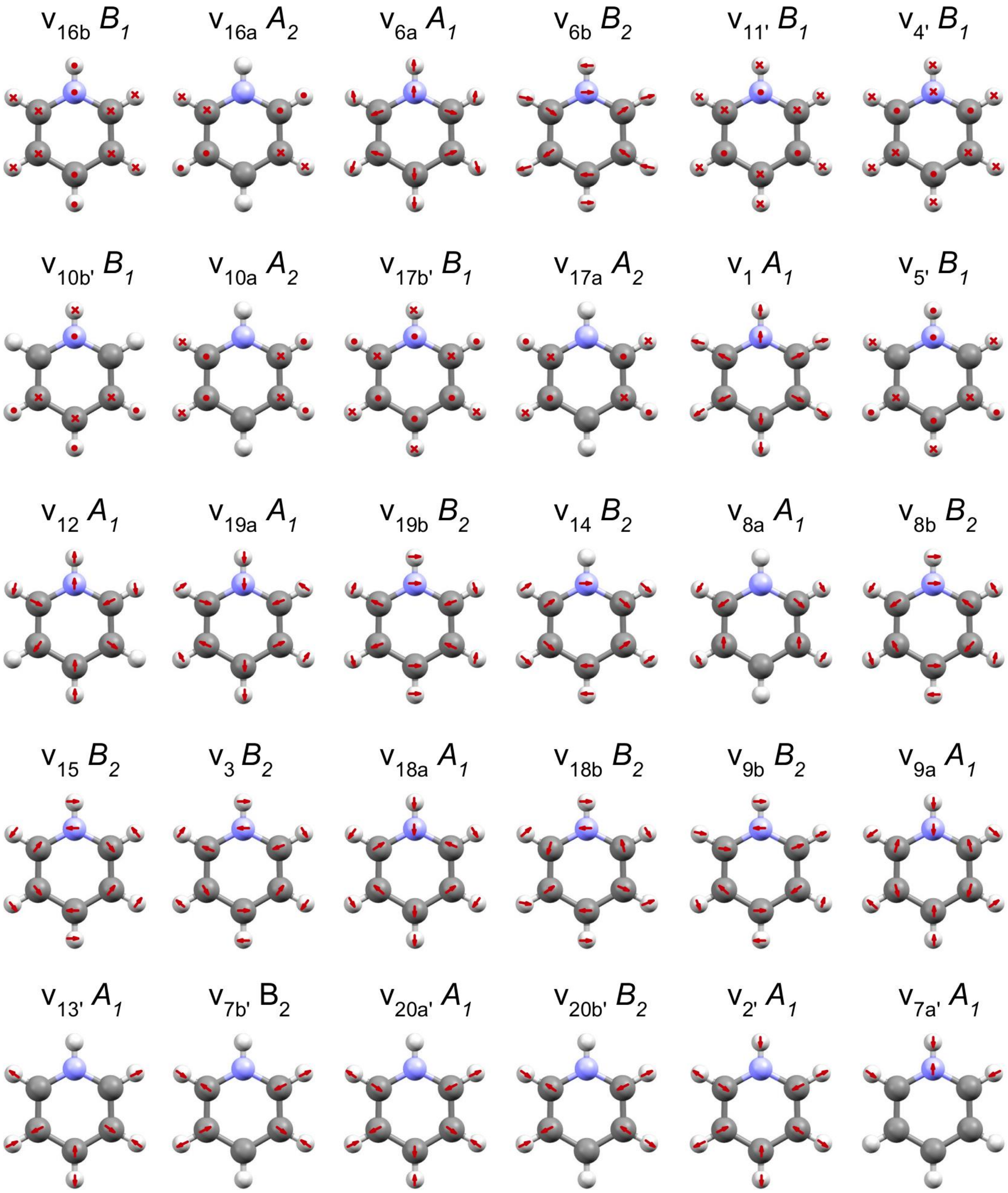

Figure S3. Calculated atomic displacements in internal normal modes of the PyH^+ cation. The notation in Wilson nomenclature and symmetry of the modes are labeled. Red crosses and dots indicate out-of-plane atomic displacements.

Table S4. Calculated anharmonic fundamental modes (from 1 to 30), overtones (Over) and combination modes (Comb) of the PyH^+ cation.

	Wilson	Freq., cm^{-1}	Intencity *I* (IR), arb.u.
1	ν_{16b}	380.823	9.039
2	ν_{16a}	393.884	0
3	ν_{6a}	616.175	0.119
4	ν_{6b}	639.931	2.395
5	$\nu_{11'}$	662.532	816.126
6	$\nu_{4'}$	743.416	214.622
7	$\nu_{10b'}$	833.925	19.379
8	ν_{10a}	867.786	0
9	$\nu_{17b'}$	974.0	2.16
10	ν_{17a}	991.774	0
11	ν_{1}	1002.416	14.377
12	$\nu_{5'}$	1037.482	2.078
13	ν_{12}	1029.52	8.18
14	ν_{19a}	1061.915	8.389
15	ν_{19b}	1050.837	11.709
16	ν_{14}	1185.524	12.625
17	ν_{8a}	1208.974	6.719
18	ν_{8b}	1266.874	6.005
19	ν_{15}	1330.21	26.858
20	ν_{3}	1389.439	11.217
21	ν_{18a}	1485.738	57.755
22	ν_{18b}	1536.821	131.531
23	ν_{9b}	1603.321	46.876
24	ν_{9a}	1624.768	60.12
25	$\nu_{13'}$	3031.568	3.641
26	$\nu_{7b'}$	3087.254	2.88
27	$\nu_{20a'}$	3066.797	4.631
28	$\nu_{20b'}$	3106.355	16.845
29	$\nu_{2'}$	3105.509	9.12
30	$\nu_{7a'}$	3391.714	167.26
Over(1)		761.545	0.19
Over(2)		787.633	0.014
Over(3)		1233.192	0.158
Over(4)		1280.378	0.011
Over(5)		1339.733	1.227
Over(6)		1473.357	1.023
Over(7)		1666.224	1.354
Over(8)		1735.248	0.655
Over(9)		1950.918	2.635
Over(10)		1982.718	0.039
Over(11)		2003.549	2.2759E-5
Over(12)		2074.805	0.819
Over(13)		2058.476	0.002
Over(14)		2123.059	0.178
Over(15)		2122.049	0.047
Over(16)		2373.364	0.709
Over(17)		2418.213	0.17
Over(18)		2533.895	0.015
Over(19)		2654.226	0.039
Over(20)		2774.339	6.915453E-4
Over(21)		2965.054	0.004

Over(22)	3089.785	0.561
Over(23)	3191.119	6.004
Over(24)	3251.483	0.53
Over(25)	6060.025	0.472
Over(26)	6099.457	0.066
Over(27)	6181.595	0.002
Over(28)	6213.532	1.397357E-4
Over(29)	6166.862	0.159
Over(30)	6640.038	2.271
Comb(1-2)	774.622	0.124
Comb(1-3)	997.629	0.274
Comb(1-4)	1020.277	0
Comb(1-5)	1050.892	0.965
Comb(1-6)	1119.208	0.089
Comb(1-7)	1214.264	1.129
Comb(1-8)	1243.327	8.962
Comb(1-9)	1354.948	0.19
Comb(1-10)	1368.591	2.056
Comb(1-11)	1384.281	0.002
Comb(1-12)	1416.888	1.266
Comb(1-13)	1406.18	0.008
Comb(1-14)	1443.087	0.018
Comb(1-15)	1441.709	0
Comb(1-16)	1565.334	0
Comb(1-17)	1589.316	0.004
Comb(1-18)	1648.717	0
Comb(1-19)	1710.409	0
Comb(1-20)	1772.329	0
Comb(1-21)	1866.99	0.032
Comb(1-22)	1919.057	0
Comb(1-23)	1980.131	0
Comb(1-24)	2005.406	0.104
Comb(1-25)	3454.33	0.004
Comb(1-26)	3474.017	0
Comb(1-27)	3469.706	3.28492E-5
Comb(1-28)	3488.65	0
Comb(1-29)	3486.202	7.078424E-4
Comb(1-30)	3772.504	0.103
Comb(2-3)	1009.713	0
Comb(2-4)	1035.122	0.252
Comb(2-5)	1074.37	7.503
Comb(2-6)	1130.333	0.018
Comb(2-7)	1221.931	1.851
Comb(2-8)	1261.084	0.247
Comb(2-9)	1369.437	0.265
Comb(2-10)	1385.209	0.494
Comb(2-11)	1397.271	0
Comb(2-12)	1429.934	0.476
Comb(2-13)	1422.897	0
Comb(2-14)	1455.459	0
Comb(2-15)	1453.821	7.317357E-4
Comb(2-16)	1579.378	4.43048E-5
Comb(2-17)	1601.677	0
Comb(2-18)	1662.268	0.006
Comb(2-19)	1722.07	0.038

Comb(2-20)	1781.577	9.160326E-4
Comb(2-21)	1878.811	0
Comb(2-22)	1933.094	0.008
Comb(2-23)	1994.027	0.176
Comb(2-24)	2017.703	0
Comb(2-25)	3467.58	0
Comb(2-26)	3487.098	0.011
Comb(2-27)	3482.795	0
Comb(2-28)	3501.451	0.006
Comb(2-29)	3499.009	0
Comb(2-30)	3785.637	0
Comb(3-4)	1255.959	0.501
Comb(3-5)	1285.704	0.001
Comb(3-6)	1352.279	8.209324E-4
Comb(3-7)	1449.322	9.729049E-4
Comb(3-8)	1483.719	0
Comb(3-9)	1591.094	0.002
Comb(3-10)	1607.594	0
Comb(3-11)	1614.429	13.305
Comb(3-12)	1652.462	7.973667E-4
Comb(3-13)	1645.913	12.304
Comb(3-14)	1677.543	0.152
Comb(3-15)	1677.722	0.219
Comb(3-16)	1801.781	0.02
Comb(3-17)	1824.892	3.935E-7
Comb(3-18)	1883.811	0.011
Comb(3-19)	1943.912	0.009
Comb(3-20)	2004.475	0.008
Comb(3-21)	2100.618	0.037
Comb(3-22)	2153.639	0.038
Comb(3-23)	2214.778	0.041
Comb(3-24)	2242.86	2.8104E-6
Comb(3-25)	3689.886	0.183
Comb(3-26)	3709.425	0.059
Comb(3-27)	3705.106	0.003
Comb(3-28)	3724.115	0.036
Comb(3-29)	3721.638	0.002
Comb(3-30)	4007.735	0.104
Comb(4-5)	1309.246	0
Comb(4-6)	1376.273	0
Comb(4-7)	1473.567	0
Comb(4-8)	1507.232	0.015
Comb(4-9)	1614.748	0
Comb(4-10)	1631.598	5.606093E-4
Comb(4-11)	1645.534	14.461
Comb(4-12)	1675.433	0
Comb(4-13)	1669.41	0.851
Comb(4-14)	1701.751	0.596
Comb(4-15)	1701.149	0.183
Comb(4-16)	1825.734	0.067
Comb(4-17)	1848.601	0.012
Comb(4-18)	1907.811	0.142
Comb(4-19)	1967.871	0.05
Comb(4-20)	2028.277	7.044219E-4
Comb(4-21)	2124.389	0.079

Comb(4-22)	2177.607	0.126
Comb(4-23)	2235.835	0.058
Comb(4-24)	2262.83	1.28524E-5
Comb(4-25)	3713.685	0.034
Comb(4-26)	3733.233	5.306006E-4
Comb(4-27)	3728.952	0.298
Comb(4-28)	3747.598	0.138
Comb(4-29)	3745.137	0.028
Comb(4-30)	4031.385	9.757207E-4
Comb(5-6)	1407.282	0.083
Comb(5-7)	1504.307	6.676
Comb(5-8)	1536.752	2.074
Comb(5-9)	1646.623	1.058
Comb(5-10)	1661.943	0.465
Comb(5-11)	1671.194	0.023
Comb(5-12)	1707.827	0.006
Comb(5-13)	1694.137	0.15
Comb(5-14)	1733.941	0.056
Comb(5-15)	1730.847	0
Comb(5-16)	1855.799	0
Comb(5-17)	1879.096	0.004
Comb(5-18)	1936.881	0
Comb(5-19)	1997.854	0
Comb(5-20)	2058.899	0
Comb(5-21)	2154.906	9.941531E-4
Comb(5-22)	2208.632	0
Comb(5-23)	2269.066	0
Comb(5-24)	2294.362	0.003
Comb(5-25)	3741.929	0.012
Comb(5-26)	3762.076	0
Comb(5-27)	3757.581	8.614471E-4
Comb(5-28)	3775.777	0
Comb(5-29)	3773.359	0.07
Comb(5-30)	4059.804	0.082
Comb(6-7)	1567.162	9.058
Comb(6-8)	1604.006	1.333
Comb(6-9)	1712.865	0.534
Comb(6-10)	1727.935	0.027
Comb(6-11)	1737.838	0.068
Comb(6-12)	1774.366	0.128
Comb(6-13)	1765.178	0.011
Comb(6-14)	1796.419	0.015
Comb(6-15)	1796.92	0
Comb(6-16)	1922.647	0
Comb(6-17)	1945.826	0.002
Comb(6-18)	2004.236	0
Comb(6-19)	2065.033	0
Comb(6-20)	2125.147	0
Comb(6-21)	2221.82	0.002
Comb(6-22)	2275.023	0
Comb(6-23)	2336.225	0
Comb(6-24)	2360.562	0.026
Comb(6-25)	3809.265	1.41918E-4
Comb(6-26)	3827.777	0
Comb(6-27)	3823.703	0.021

Comb(6-28)	3843.097	0
Comb(6-29)	3840.564	0.058
Comb(6-30)	4124.558	0.32
Comb(7-8)	1702.113	0.399
Comb(7-9)	1809.182	5.2
Comb(7-10)	1824.896	1.058
Comb(7-11)	1835.03	0.003
Comb(7-12)	1868.789	0.105
Comb(7-13)	1862.43	0.031
Comb(7-14)	1894.915	0.026
Comb(7-15)	1895.526	0
Comb(7-16)	2020.24	0
Comb(7-17)	2042.548	0.011
Comb(7-18)	2102.099	0
Comb(7-19)	2166.164	0
Comb(7-20)	2224.368	0
Comb(7-21)	2319.27	0.008
Comb(7-22)	2372.906	0
Comb(7-23)	2431.76	0
Comb(7-24)	2455.798	0.019
Comb(7-25)	3905.539	0.008
Comb(7-26)	3925.563	0
Comb(7-27)	3921.228	0.056
Comb(7-28)	3940.831	0
Comb(7-29)	3938.31	0.03
Comb(7-30)	4213.679	0.588
Comb(8-9)	1840.652	2.385
Comb(8-10)	1856.93	5.312
Comb(8-11)	1869.538	0
Comb(8-12)	1903.407	1.007
Comb(8-13)	1896.66	0
Comb(8-14)	1929.592	0
Comb(8-15)	1929.21	0.095
Comb(8-16)	2053.658	0.01
Comb(8-17)	2077.823	0
Comb(8-18)	2136.353	0.073
Comb(8-19)	2199.798	0.025
Comb(8-20)	2257.582	0.007
Comb(8-21)	2353.51	0
Comb(8-22)	2406.695	0.016
Comb(8-23)	2467.796	0.011
Comb(8-24)	2491.828	0
Comb(8-25)	3940.779	0
Comb(8-26)	3957.465	0.011
Comb(8-27)	3953.503	0
Comb(8-28)	3971.098	0.231
Comb(8-29)	3968.675	0
Comb(8-30)	4258.824	0
Comb(9-10)	1966.494	3.778
Comb(9-11)	1976.533	3.732795E-4
Comb(9-12)	2012.023	0.494
Comb(9-13)	2004.567	0.024
Comb(9-14)	2036.68	0.032
Comb(9-15)	2036.29	0
Comb(9-16)	2160.832	0

Comb(9-17)	2183.948	0.008
Comb(9-18)	2241.629	0
Comb(9-19)	2303.441	0
Comb(9-20)	2364.247	0
Comb(9-21)	2460.97	0.005
Comb(9-22)	2513.673	0
Comb(9-23)	2574.923	0
Comb(9-24)	2598.639	0.055
Comb(9-25)	4047.438	0.007
Comb(9-26)	4066.35	0
Comb(9-27)	4061.692	0.155
Comb(9-28)	4077.259	0
Comb(9-29)	4075.086	0.101
Comb(9-30)	4362.859	0.089
Comb(10-11)	1993.185	0
Comb(10-12)	2028.578	1.513
Comb(10-13)	2020.947	0
Comb(10-14)	2053.579	0
Comb(10-15)	2052.607	0.006
Comb(10-16)	2177.47	0.03
Comb(10-17)	2200.402	0
Comb(10-18)	2259.022	0.131
Comb(10-19)	2320.655	0.112
Comb(10-20)	2384.426	0.015
Comb(10-21)	2477.327	0
Comb(10-22)	2530.331	0.004
Comb(10-23)	2591.714	0.037
Comb(10-24)	2615.414	0
Comb(10-25)	4064.495	0
Comb(10-26)	4079.888	0.25
Comb(10-27)	4076.365	0
Comb(10-28)	4095.393	0.016
Comb(10-29)	4092.842	0
Comb(10-30)	4383.047	0
Comb(11-12)	2037.777	0.002
Comb(11-13)	2031.116	0.024
Comb(11-14)	2063.226	0.042
Comb(11-15)	2063.009	0.019
Comb(11-16)	2187.254	0.038
Comb(11-17)	2210.606	0.039
Comb(11-18)	2267.682	0.032
Comb(11-19)	2328.094	0.126
Comb(11-20)	2390.267	0.017
Comb(11-21)	2486.268	0.054
Comb(11-22)	2539.021	0.044
Comb(11-23)	2596.866	0.017
Comb(11-24)	2627.958	0.053
Comb(11-25)	4076.493	0.007
Comb(11-26)	4095.579	0.139
Comb(11-27)	4091.373	0.016
Comb(11-28)	4110.439	0.015
Comb(11-29)	4107.926	0.013
Comb(11-30)	4393.6	0.062
Comb(12-13)	2065.484	0.034
Comb(12-14)	2097.9	0.008

Comb(12-15)	2097.826	0
Comb(12-16)	2221.765	0
Comb(12-17)	2245.41	0.004
Comb(12-18)	2304.332	0
Comb(12-19)	2365.168	0
Comb(12-20)	2424.815	0
Comb(12-21)	2522.48	0.001
Comb(12-22)	2575.223	0
Comb(12-23)	2636.181	0
Comb(12-24)	2661.218	0.026
Comb(12-25)	4100.357	0.234
Comb(12-26)	4126.283	0
Comb(12-27)	4121.194	0.011
Comb(12-28)	4143.182	0
Comb(12-29)	4140.423	0.009
Comb(12-30)	4427.816	1.071632E-4
Comb(13-14)	2090.571	0.007
Comb(13-15)	2091.319	2.023199E-4
Comb(13-16)	2214.774	0.007
Comb(13-17)	2237.818	0.153
Comb(13-18)	2296.51	0.059
Comb(13-19)	2356.466	0.003
Comb(13-20)	2417.463	0.182
Comb(13-21)	2513.249	0.009
Comb(13-22)	2567.489	0.066
Comb(13-23)	2627.466	0.146
Comb(13-24)	2650.97	0.018
Comb(13-25)	4102.104	0.414
Comb(13-26)	4122.409	0.321
Comb(13-27)	4117.95	0.033
Comb(13-28)	4136.298	0.053
Comb(13-29)	4133.846	0.26
Comb(13-30)	4420.192	0.341
Comb(14-15)	2123.052	0.076
Comb(14-16)	2247.592	0.067
Comb(14-17)	2269.779	0.096
Comb(14-18)	2328.284	0.104
Comb(14-19)	2390.049	0.002
Comb(14-20)	2449.843	0.615
Comb(14-21)	2545.464	0.014
Comb(14-22)	2599.13	0.084
Comb(14-23)	2657.914	0.117
Comb(14-24)	2684.797	0.127
Comb(14-25)	4135.475	3.2413E-6
Comb(14-26)	4152.366	0.549
Comb(14-27)	4148.621	0.01
Comb(14-28)	4167.926	0.09
Comb(14-29)	4165.4	0.037
Comb(14-30)	4453.892	0.026
Comb(15-16)	2246.579	0.325
Comb(15-17)	2270.827	0.86
Comb(15-18)	2327.789	0.016
Comb(15-19)	2389.703	0.164
Comb(15-20)	2449.071	1.268
Comb(15-21)	2545.977	0.019

Comb(15-22)	2597.812	1.229E-7
Comb(15-23)	2659.407	0.096
Comb(15-24)	2681.798	0.035
Comb(15-25)	4132.628	0.041
Comb(15-26)	4153.957	0.143
Comb(15-27)	4149.265	0.002
Comb(15-28)	4168.412	0.053
Comb(15-29)	4165.819	0.037
Comb(15-30)	4451.601	0.07
Comb(16-17)	2395.982	0.917
Comb(16-18)	2454.277	0.011
Comb(16-19)	2513.49	0.149
Comb(16-20)	2570.742	0.059
Comb(16-21)	2668.659	0.284
Comb(16-22)	2721.188	0.097
Comb(16-23)	2784.159	0.048
Comb(16-24)	2808.962	0.052
Comb(16-25)	4252.957	0.035
Comb(16-26)	4275.354	0.053
Comb(16-27)	4270.914	0.003
Comb(16-28)	4292.389	0.005
Comb(16-29)	4289.545	0.005
Comb(16-30)	4577.05	0.008
Comb(17-18)	2476.279	0.193
Comb(17-19)	2536.888	0.113
Comb(17-20)	2596.175	1.92388E-5
Comb(17-21)	2691.526	0.562
Comb(17-22)	2747.027	0.272
Comb(17-23)	2808.532	0.028
Comb(17-24)	2830.038	0.198
Comb(17-25)	4282.098	0.038
Comb(17-26)	4298.462	0.021
Comb(17-27)	4294.631	0.102
Comb(17-28)	4312.02	0.104
Comb(17-29)	4310.095	0.001
Comb(17-30)	4600.566	5.423092E-4
Comb(18-19)	2597.274	3.948
Comb(18-20)	2655.467	0.451
Comb(18-21)	2751.365	0.296
Comb(18-22)	2803.746	0.253
Comb(18-23)	2863.424	1.189
Comb(18-24)	2890.104	0.182
Comb(18-25)	4341.703	0.029
Comb(18-26)	4360.661	5.92372E-5
Comb(18-27)	4356.037	0.037
Comb(18-28)	4372.684	0.01
Comb(18-29)	4370.278	0.096
Comb(18-30)	4654.874	0.111
Comb(19-20)	2715.32	0.033
Comb(19-21)	2808.994	0.333
Comb(19-22)	2858.044	0.012
Comb(19-23)	2921.646	2.15
Comb(19-24)	2941.427	1.414
Comb(19-25)	4405.884	0.004
Comb(19-26)	4423.556	0.005

Comb(19-27)	4420.039	0.07
Comb(19-28)	4441.824	0.006
Comb(19-29)	4436.175	0.103
Comb(19-30)	4720.613	0.083
Comb(20-21)	2867.842	0.429
Comb(20-22)	2924.043	1.12
Comb(20-23)	2981.688	2.085
Comb(20-24)	3004.891	0.538
Comb(20-25)	4461.15	0.006
Comb(20-26)	4486.199	0.049
Comb(20-27)	4477.545	0.034
Comb(20-28)	4495.914	0.113
Comb(20-29)	4493.184	0.005
Comb(20-30)	4777.065	0.139
Comb(21-22)	3019.41	0.133
Comb(21-23)	3077.544	0.822
Comb(21-24)	3151.231	4.265
Comb(21-25)	4558.583	2.00144E-5
Comb(21-26)	4584.475	0.084
Comb(21-27)	4571.134	0.011
Comb(21-28)	4595.79	0.015
Comb(21-29)	4595.59	0.107
Comb(21-30)	4876.706	0.019
Comb(22-23)	3131.982	0.166
Comb(22-24)	3172.483	1.572
Comb(22-25)	4612.028	5.882E-7
Comb(22-26)	4628.94	0.012
Comb(22-27)	4628.776	0.03
Comb(22-28)	4646.442	0.03
Comb(22-29)	4650.344	0.011
Comb(22-30)	4927.522	0.13
Comb(23-24)	3226.052	0.167
Comb(23-25)	4676.725	0.029
Comb(23-26)	4695.073	0.026
Comb(23-27)	4688.781	0.209
Comb(23-28)	4708.69	0.078
Comb(23-29)	4707.41	0.072
Comb(23-30)	5000.851	0.057
Comb(24-25)	4697.552	0.114
Comb(24-26)	4718.921	0.005
Comb(24-27)	4712.418	0.005
Comb(24-28)	4737.531	0.277
Comb(24-29)	4720.695	0.046
Comb(24-30)	5014.712	0.173
Comb(25-26)	6155.54	0.186
Comb(25-27)	6128.714	0.01
Comb(25-28)	6178.531	0.003
Comb(25-29)	6173.307	0.008
Comb(25-30)	6465.708	0.003
Comb(26-27)	6121.268	0.739
Comb(26-28)	6168.959	0.163
Comb(26-29)	6163.959	0.003
Comb(26-30)	6485.076	2.644044E-4
Comb(27-28)	6164.296	0.029
Comb(27-29)	6159.911	0.197

Comb(27-30)	6480.81	0.006
Comb(28-29)	6141.352	0.65
Comb(28-30)	6500.101	0.004
Comb(29-30)	6497.497	0.019

Table S5. Combination modes recognized in the experimental vibrational spectra.

	PyHI	PyHBr	$PyPbI_3$	$PyPbBr_3$
	Freq., cm-1			
$\nu_{16a+}\nu_{6b}$	1034	-	1034	1038
$\nu_{16a}+\nu_{11'}$	1077	1088	1062	1065
$\nu_{16b}+\nu_{10b'}$	1264	1278	1224	1233
$\nu_{16b}+\nu_{17a}$	-	-	1356	1355
$\nu_{11'}+ \nu_{4'}$	1423	1430	1385	1394
$\nu_{16b}+\nu_{5'}$	-	-	1394	1404
$\nu_1+\nu_{6a}$	1609	-	1611	1612
$\nu_1+\nu_{6b}$	1643	-	1642	-
$\nu_{12}+\nu_{6a}$	1634	-	-	-

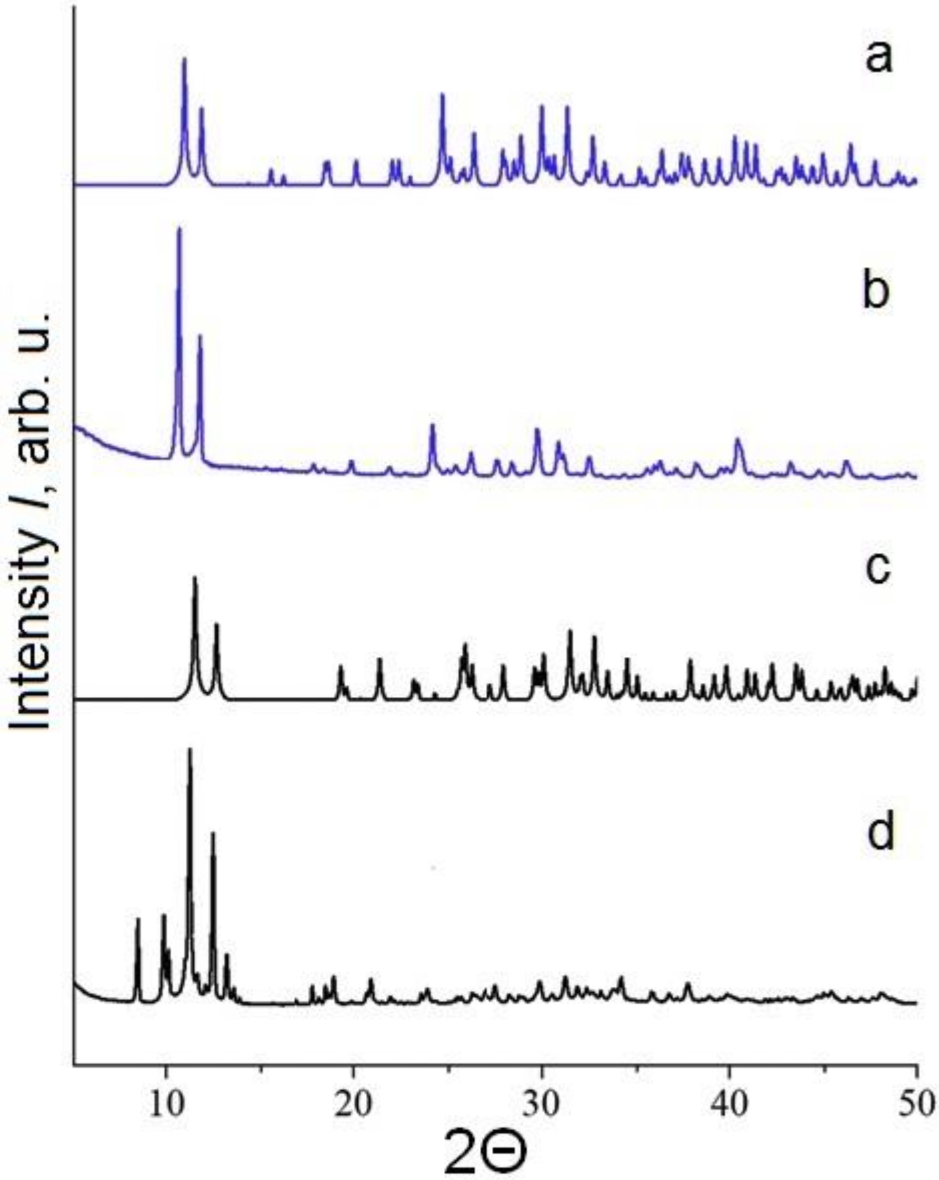

Figure S4. Powder XRD patterns simulated from the single crystal data (a,c) and measured for grounded single crystals (b,d) for $PyPbI_3$ (a,b) and $PyPbBr_3$ (c,d).

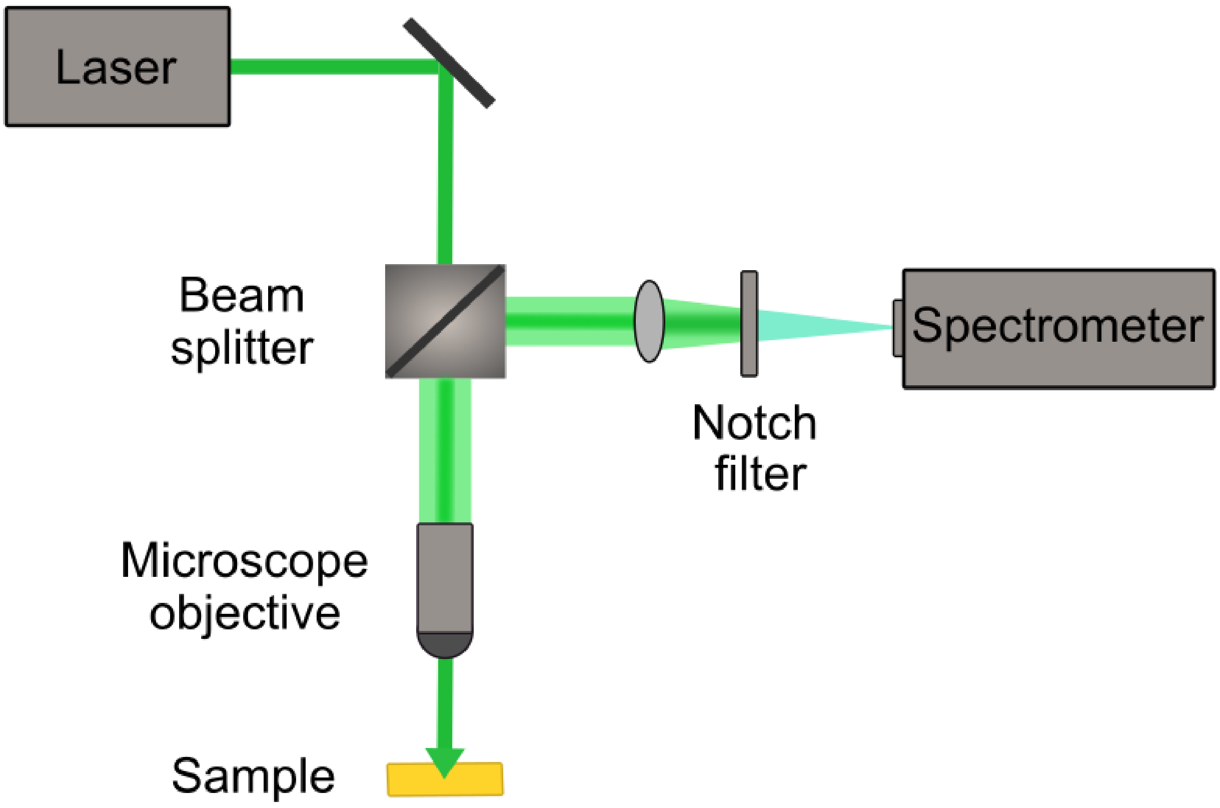

Figure S5. The scheme of the Raman measurement experiment.
